# Evaluation of the Impact of Infusion Set Design on the Particulate Load Induced by Vancomycin–Piperacillin/Tazobactam Incompatibility

**DOI:** 10.3390/ph17091222

**Published:** 2024-09-17

**Authors:** Laura Négrier, Bertrand Décaudin, Anthony Treizebré, Marie Guilbert, Pascal Odou, Anthony Martin Mena

**Affiliations:** 1 University of Lille, CHU Lille, ULR 7365—GRITA—Groupe de Recherche sur les formes Injectables et les Technologies Associées, F-59000 Lille, France; laura.negrier@univ-lille.fr (L.N.); bertrand.decaudin@univ-lille.fr (B.D.); pascal.odou@univ-lille.fr (P.O.); 2 University of Lille, CNRS, Centrale Lille, University Polytechnique Hauts-de-France, UMR 8520—IEMN—Institut d’Electronique de Microélectronique et de Nanotechnologie, F-59000 Lille, France; anthony.treizebre@univ-lille.fr (A.T.); marie.guilbert@univ-lille.fr (M.G.)

**Keywords:** vancomycin, piperacillin/tazobactam, drug incompatibility, infusion, particulate load, common volume, multilumen device

## Abstract

Introduction: Drug incompatibilities are among the most common medication errors in intensive care units. A precipitate can form and block the catheter or cause an adverse event in the patient. Intensive care units have implemented various strategies for limiting the occurrence of these incompatibilities, which have already been studied in vitro under standardized conditions. The objective of the present in vitro study was to continue these assessments by determining the impact of the infusion line geometry and the drugs’ position in the infusion set-up on the prevention of vancomycin–piperacillin/tazobactam incompatibility. Methods: Infusion lines with a different common volume, a multilumen medical infusion device, a dilute vancomycin solution, and separate infusions of incompatible drugs were evaluated separately. The infusion line outlet was connected to a dynamic particle counter. Results: Reducing the common volume, using multilumen medical devices, or spacing out the two incompatible drugs on the infusion line did not prevent the occurrence of a significant particulate load. Only dilution of the vancomycin solution was associated with a significantly lower particulate load and the absence of drug incompatibility. Conclusions: Our results show that under specific conditions, it is possible to reduce particulate contamination considerably.

## 1. Introduction

Intensive care units are faced with the risk of drug incompatibilities, which can account for up to 25% of medication errors [1]. Half of the incompatibilities observed in intensive care units concern anti-infectives, such as cephalosporins and vancomycin [2,3,4,5]. These drug incompatibilities are physicochemical reactions that may result in the formation of a precipitate, i.e., clusters of particles that may or may not be visible to the naked eye [6]. This precipitate can block the infusion lines and lead to adverse events in patients, ranging from thrombosis and phlebitis to organ dysfunction [7,8,9,10]. It is, therefore, essential for intensive care units to prevent and mitigate the occurrence of drug incompatibility.

Over the last few years, care services have implemented various strategies for dealing with the issue of drug incompatibility [11]. Firstly, medical staff can refer to and use compatibility databases, double-entry cross-tabulations, and/or the literature data to find out whether or not two injectable drugs are likely to be incompatible (e.g., Stabilis and the King Guide) [12,13,14,15]. Unfortunately, staff often lack knowledge about the risk of drug incompatibility. Furthermore, the drug compatibility data are often missing or contradictory, which increases the likelihood of an incident [1,16,17]. Secondly, a number of clinical strategies may avoid or limit contact between incompatible drugs. The use of separate infusion lines or sequential infusions can be considered. However, a limited number of venous access points or the need for continuous infusion of certain drugs can restrict these approaches [18]. For these reasons, the most common approach involves limiting the contact time between drug solutions. Departments will, therefore, implement standard operating procedures or change practices by (i) using a particular design for the infusion line, (ii) minimizing drug concentrations, and/or (iii) using multilumen infusion devices to limit drug contact time. According to the literature data, decreasing an infusion line’s common volume (defined as the volume between the point where the drug and inert carrier streams meet and the catheter’s inlet) can reduce the contact time and, thus, the likelihood of particle formation [19,20,21]. In-line filtration is also a potential solution for preventing the administration of an incompatibility-related particulate load. However, some drugs interact with the filtration membrane, so this approach is not always applicable [11]. Hence, a multimodal combination of strategies is often required to reduce the risk of incompatibility [18,19].

The combination of vancomycin and piperacillin/tazobactam (VPT) is frequently prescribed in intensive care units [22,23]. This empirical first-line combination treatment is recommended in several clinical guidelines for the treatment of serious infections [24,25,26]. The VPT combination’s degree of (in)compatibility depends mainly on the concentration of the vancomycin (V) solution [18,27]. Researchers have evaluated the strategies used in clinical wards to limit the risk of drug incompatibility for standard doses of intravenously administered VPT [18,28]. These studies identified a number of factors that influence the particulate load and the dose potentially administered to the patient: the tubing’s common volume, the flow rate of the hydration volume support, and the V concentration. The best strategy for reducing the particulate load involved lowering the V concentration and increasing the V infusion rate. However, these studies did not investigate the impact of infusion parameters on the risk of drug precipitate formation when two drugs can be administered simultaneously on the same infusion line: the length and diameter of the infusion line and the drugs’ respective positions in the infusion set-up.

Hence, the primary objective of this in vitro study was to determine the impact of the infusion line geometry and the drugs’ position in the infusion set-up on VPT incompatibility with standard and low-concentration V solutions.

## 2. Results

### 2.1. The Overall Results

**Figure 1:**
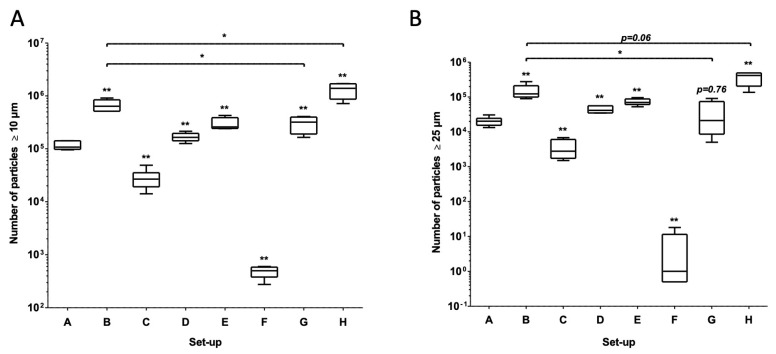
Impact of the type of infusion set-up (set-ups A, B, C, D, E, F, G, and H) on the particle count (particles ≥ 10 µm in (**A**) and particles ≥ 25 µm in (**B**)) during the 4.5 h simulated infusion. The results are expressed as the median (range). In a Mann–Whitney test, set-ups B to H were compared with the standard set-up (set-up A): * *p* < 0.05 and ** *p* < 0.01 (n = 5). In a Mann–Whitney test, set-ups G and H were compared with set-up B: * *p* < 0.05 (n = 5).

In the infusion set-ups tested here, the total particle count and the counts of particles ≥ 10 µm and ≥25 µm varied according to the tubing’s length, internal diameter, and common volume (Table 1 and Figure 1A,B). Set-up F (V diluted to 5.95 mg/mL, with a three-port manifold 200 cm in length and 2.5 mm in diameter) had the lowest mean ± standard deviation (SD) number of particles ≥ 10 µm (485 ± 125) and ≥25 µm (5 ± 8). Set-up H (V infusion placed 150 cm downstream of the manifold, i.e., 50 cm upstream of the outlet of the infusion line) had the highest mean ± SD number of particles ≥ 10 µm (1,306,725 ± 431,423) and ≥25 µm (361,961 ± 154,142) (Table 1). 

The particle count observed over the course of the 4.5 h simulated infusion was broken down into several parts for analysis. Two periods were analyzed in particular. Firstly, a period referred to hereafter as “peak 1” corresponded to the first major period of particle release in most of the eight set-ups studied here. According to the plug-flow model, peak 1 corresponded to the time just before the equilibration of the mixture at the outlet of the extension set after the piperacillin/tazobactam (PT) syringe had been turned on [29]. Secondly, “peak 2” corresponded to the second major period of particle release in some of the eight set-ups studied, i.e., just before a homogeneous mixture was obtained at the outlet of the extension set after the PT syringe had been turned off. The peak durations varied from one set-up to another, with mean ± SD values ranging from 5 ± 0 to 9 ± 2 min for peak 1 and from 6 ± 2.5 to 25 ± 4.5 min for peak 2 (Table 2).

**Figure 2:**
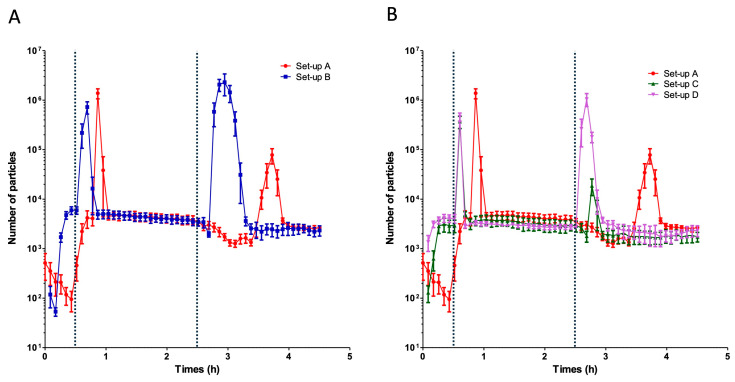
Influence of the length and internal diameter of the extension set on the total particle count during the infusion for (**A**) set-up A (in red) and set-up B (in blue), (**B**) set-up A (in red), set-up C (in dark green), and set-up D (in pale purple). The dotted lines correspond to the start and the end of the PT infusion (t = 30 min and t = 2.5 h, respectively). The results are expressed as the mean ± SD, n = 5.

**Figure 3:**
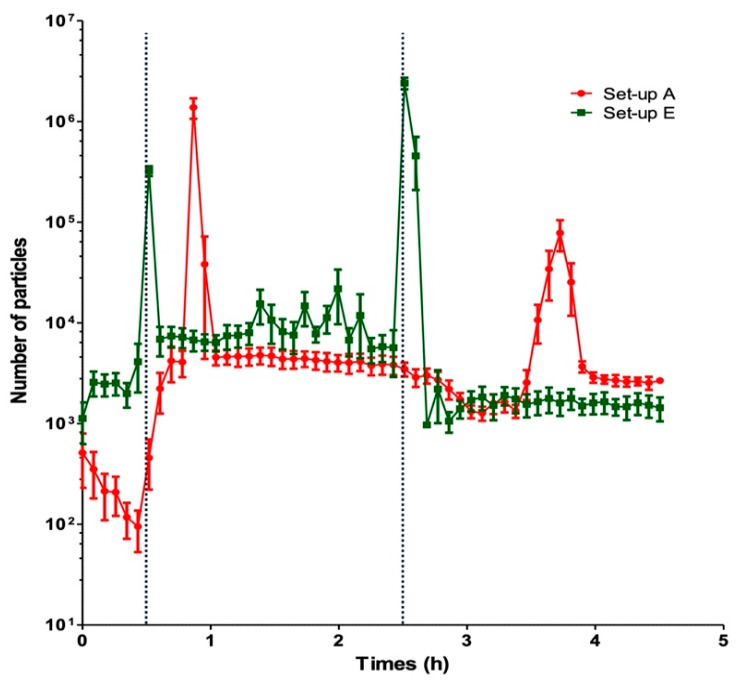
Influence of the use of a multilumen infusion device on the particle count. The change over time in the particle count is shown for the standard set-up A (in red) and the multilumen set-up E (in green). The dotted lines correspond to the start and the end of the PT infusion (t = 30 min and t = 2.5 h, respectively). The results are expressed as the mean ± SD, n = 5.

**Figure 4:**
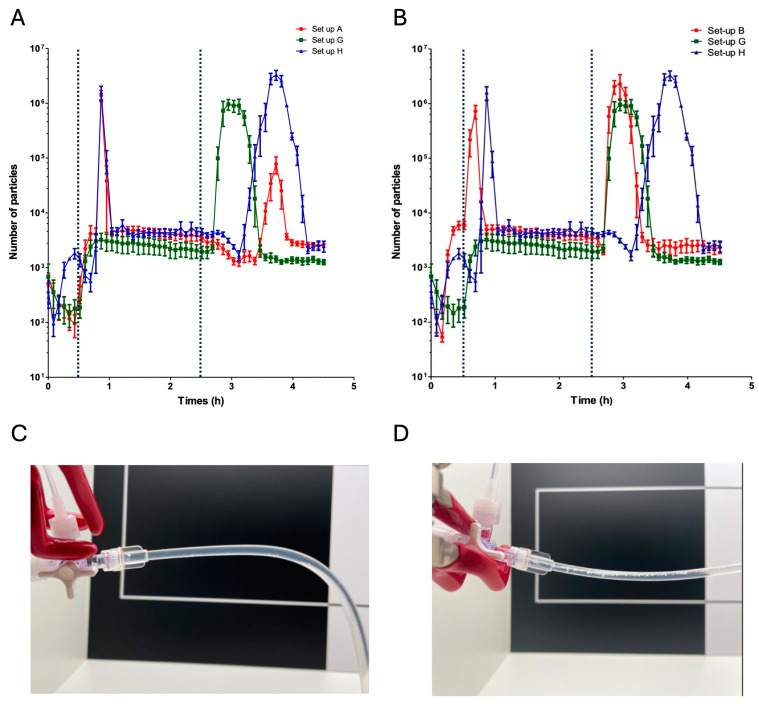
Influence of the length of tubing between the vancomycin line and the PT line on the particle count. (**A**) The particle count as a function of the infusion time for set-up A (in red), set-up G (in green), and set-up H (in blue). (**B**) The particle count as a function of the infusion time for set-up B (in red), set-up G (in green), and set-up H (in blue). The dotted lines correspond to the start and the end of the PT infusion (t = 30 min and t = 2.5 h, respectively). The results are expressed as the mean ± standard deviation, n = 5. (**C**,**D**) Visual observation of the infusion lines. The absence of visible precipitate during a VPT co-infusion in the set-up G (**C**). The presence of visible precipitate during a VPT co-infusion in the set-up H (**D**).

**Figure 5:**
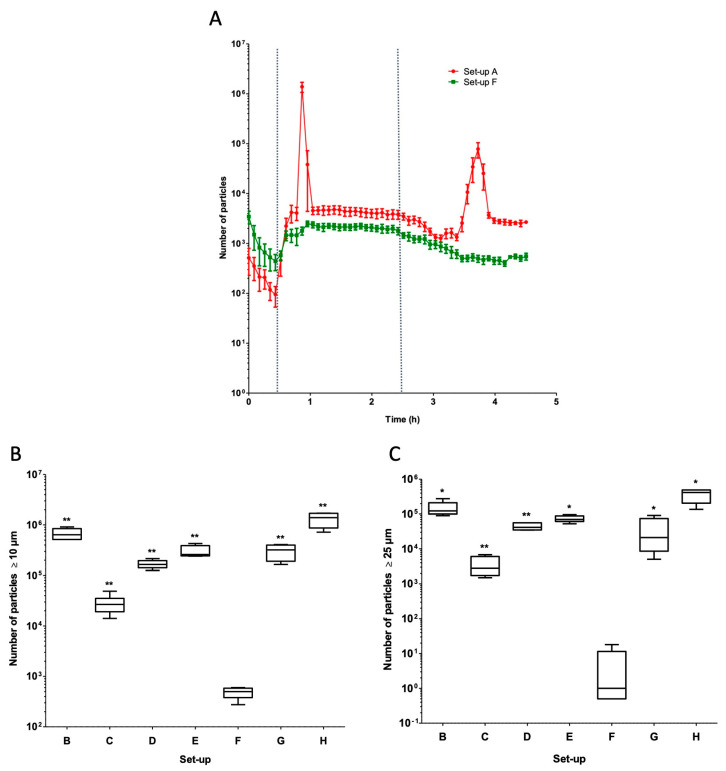
(**A**) Influence of the V solution concentration on the particle count as a function of the infusion time for set-ups A (in red) and F (in green). The dotted lines correspond to the start and the end of the PT infusion (t = 30 min and t = 2.5 h, respectively). The results are expressed as the mean ± standard deviation, n = 5. (**B**,**C**) Impact of the choice of infusion set or protocol on the particle count. Comparisons of the particle count ≥ 10 µm (**B**) and the particle count ≥ 25 µm (**C**) in the various infusion sets and protocols (set-ups B, C, D, E, F, G, and H). All the set-ups were compared with the set-up F (containing 5.95 mg/mL V). The results are expressed as the median (range). * *p* < 0.05 and ** *p* < 0.01 in a Mann–Whitney test, n = 5.

The particle counts (total, ≥10 µm and ≥25 µm) in peak 1 and peak 2 over the course of the infusion were then summed. Peaks 1 and 2 accounted for the majority of the particles observed during the infusion (Table 1). Six of the eight set-ups (A, B, C, D, E, and H) showed the two-particle peaks characteristic of VPT incompatibility (Figure 2, Figure 3, Figure 4 and Figure 5). The number of ≥10 µm and ≥25 µm particles in peaks 1 and/or 2 differed from one set-up to another (Table 1).

### 2.2. Impact of Differences in Tubing Length and Internal Diameter

We used set-ups A to D to study the impact of the length and the internal diameter of the extension set on the particle count. Significant higher loads of particles ≥ 10 µm and particles ≥ 25 µm were observed in set-up B (tubing length: 50 cm, small internal diameter (Ø)) and set-up D (50 cm, small Ø) than in set-up A (200 cm, large Ø). Significant lower loads of particles ≥10 µm and particles ≥25 µm were observed in set-up C (200 cm, small Ø) than in set-up A (200 cm, large Ø) (Figure 1 and Table 1).

### 2.3. Impact of Using a Multilumen Medical Device

The number of particles observed with the multilumen set-up (set-up E) was compared with that observed with the standard infusion set-up A. The two characteristic particle peaks of VPT incompatibility were observed with the multilumen set-up (Figure 3) and appeared earlier because the common volume was negligible. The counts of particles ≥ 10 µm and particles ≥ 25 µm were significantly higher in set-up E than in set-up A (Figure 1 and Table 1).

### 2.4. Impact of the Location of the Infusion Site for Vancomycin and Piperacillin/Tazobactam

We used set-ups A, B, G, and H to assess the putative influence of the location of the V and PT syringes on the particle count during the infusion. No visible particles were observed at the outlet of the three-way stopcock in set-up G during the co-infusion of V and PT (Figure 4C). In contrast, visible particles were observed at the outlet of the three-way stopcock in the set-up H during the co-infusion of V and PT (Figure 4D).

The count of particles ≥ 10 µm was significantly higher in set-up G than in set-up A. Set-ups A and G did not differ significantly with regard to the count of particles ≥ 25 µm (Figure 1 and Table 1). The counts of particles ≥ 10 µm and particles ≥ 25 µm were significantly higher in set-up H than in set-up A (Figure 1 and Table 1). The counts of particles ≥ 10 µm and particles ≥ 25 µm were significantly lower in set-up G than in set-up B (Figure 1 and Table 1). The counts of particles ≥ 10 µm and particles ≥ 25 µm were significantly higher in set-up H than in set-ups A and G (Figure 1 and Table 1). The count of particles ≥ 10 µm was significantly higher in set-up H than in set-up B. Set-ups B and H did not differ significantly with regard to the count of particles ≥ 25 µm (Figure 1 and Table 1).

### 2.5. Impact of Vancomycin Dilution

The set-up F with diluted V (5.95 mg/mL) was compared with the standard set-up A with standard V (20.8 mg/mL). During infusion of the diluted V solution (set-up F), no particulate peaks were observed at the manifold or in the tubing (Figure 5A). Significantly higher counts of particles ≥ 10 µm and particles ≥ 25 µm were observed in set-up A (containing 20.8 mg/mL V), relative to set-up F (Figure 5A and Table 1).

Relative to set-up F, significantly higher counts of particles ≥ 10 µm and particles ≥ 25 µm were observed in set-ups B (50 cm, large Ø), C (200 cm, small Ø), D (50 cm, small Ø), E (the multilumen set-up), G, and H (Figure 5B,C and Table 1).

## 3. Discussion

In this in vitro study, we continued to evaluate the influence of different strategies used in healthcare institutions to limit the occurrence of drug incompatibilities. We again worked with VPT co-infusion, which is well known to produce incompatibility [28,30,31,32]. 

The physical manifestations of vancomycin–piperacillin/tazobactam incompatibility are well documented and have been extensively described in the literature. However, the precise mechanism underlying these manifestations of incompatibility has not been determined. We are now using chemical analytical techniques to try to understand the cause of this incompatibility and the nature of this precipitate.

Furthermore, various researchers have shown that this incompatibility is (i) not pH-dependent, (ii) reversible at low concentrations, and (iii) influenced by the type of diluent [18,27,30,33]. Moreover, this incompatibility is concentration-dependent (the higher the concentration, the greater the incompatibility), and the precipitate forms rapidly (rather than slowly over time) [18,28]. Static conditions (1:1 mixing in test tubes) and dynamic conditions (real Y perfusion reproduced in vitro) can give contradictory results. The in vitro duration of the infusion (4.5 h compared with 9 h in the clinic) had no impact on the occurrence of particulate peaks and very little impact on the particulate load. We had previously looked at different strategies, such as the position of the hydration volume support, the presence of an in-line filter, and the dilution of one of the drugs [18]. We found that modification of the V concentration and infusion flow rate was the best strategy for avoiding drug incompatibilities and a high particulate load. The V concentration chosen was similar to that recommended in the French summary of product characteristics [34]. Many in vitro studies have highlighted the benefits of using a V solution below a concentration of 5 mg/mL [35,36,37]. However, this type of strategy requires changes in patient management by nursing staff, with the use of an infusion bag rather than a syringe.

In the present study, we focused on the medical device and its geometry, including the internal volumes. Other researchers have already looked at the geometry of medical infusion devices [38]. Moss et al. investigated the drug delivery dynamics of a conventional tap manifold and a micro-infusion manifold designed to minimize the dead volume. They found that with a conventional tap manifold, port selection significantly affected drug delivery dynamics for continuous infusions. Despite this finding, the impact of the medical device on drug incompatibility is poorly understood. The few literature studies on this issue are insufficient [19]. Many variables appeared to be involved. For example, the common volume is known to influence the particle generation resulting from drug incompatibilities [19,20,21]. This is why multilumen devices are recommended to avoid excessive contact time between incompatible drugs and, therefore, an increase in particle generation. Perez et al. observed that the particulate load was significantly lower (but not zero) with a multilumen device than with a stopcock manifold [19].

Our results showed that the geometry of medical devices has a significant impact on the amplitude of the particle count generated by drug incompatibility. On the one hand, we found that reducing the common volume may have an influence (although not necessarily a positive one) on the particle count. On the other hand, we found that separating infusions of incompatible drugs on an infusion line (by mixing concentrated drug solutions close to the catheter) is not necessarily a good strategy for limiting the occurrence of this drug incompatibility.

### 3.1. Impact of the Common Volume of a Medical Infusion Device (Length, Internal Diameter, Multilumen Devices)

We tested parameters that are specific to medical infusion devices, namely the length of the tubing after the tap manifold (within which the drugs to be infused are mixed) and this tubing’s internal diameter. By adjusting these two parameters, we obtained set-ups with different common volumes. Although we found that the common volume does influence the particulate load likely to be administered to the patient, our detailed results contradict the literature data [19].

Changes in the length and/or diameter of the extension set (and, therefore, in the common volume) did not prevent particle formation or the onset of drug incompatibility. The two characteristic particle peaks in VPT incompatibility were found in set-ups A, B, C, and D. The two peaks appeared just after the start of the co-infusion and at the end of the co-infusion, i.e., when the flow rate changed. Abrupt changes in flow rate are known to create particle boluses [28,39]. In view of the particle counts that we observed in peaks 1 and 2, a change in flow rate cannot fully account for these features. These peaks did not appear when a dilute V solution was used at the same flow rate; hence, lowering the V concentration is still a better way of limiting VPT drug incompatibility than reducing the infusion device’s common volume.

For a given internal diameter, the particle count fell significantly as the volume increased. The length of the tubing mainly influenced the shape and size of peak 2 in several respects. The peaks that were higher and broader had significantly greater counts of particles ≥10 µm and ≥25 µm when the tubing diameter was small. One explanation for these results is that the precipitation (which forms instantaneously) is reversible, as pointed out by Nichols et al. [40]. A larger common volume would promote the dissolution of any formed particles. This hypothesis is supported by the results of our previous work, in which particles visible at the start of the tubing (at the outlet of the tap ramp) were no longer visible at the outlet of the 200 centimeter-long tubing [18].

This observation suggested that the length of the tubing is a parameter that favors partial dissolution of the precipitate. This might explain why set-ups with long tubing have significantly fewer particles ≥ 10 µm and ≥25 µm than set-ups with short tubing (for the same internal diameter). 

The use of a multilumen device also failed to avoid incompatibility or reduce the particle count. Under our conditions and the medical device used here, we observed the presence of two particulate peaks and a significant increase in the particle count. This contradicts Perez et al.’s findings in a study of the same drug incompatibility. This disparity might be due to differences in the drug concentrations, flow rates, and/or diluents used [20,33]. The fact that peak 2 was much larger with the multilumen device than with the standard set-up confirmed a dependency on (or at least an influence of) the common volume.

The strategy in which the common volume is decreased by mixing concentrated drug solutions close to the catheter does not appear to be optimal. Again, our results showed that dilution of the V solution is a better strategy than the use of a multilumen infusion device.

### 3.2. Impact of the Distance between Incompatible Drugs on an Infusion Line

We next sought to assess the impact of moving the V and PT infusions further apart on the infusion line; this increased the volume and prolonged the time during which one of the two drugs was in contact with the hydration fluid. This strategy is used in some care units to dilute one of the incompatible drugs with the other infused treatments before it comes into contact with the incompatible drug. In the case of VPT incompatibility, some departments at Lille University Medical Center infuse V close to the patient, and the other drugs are infused further up the infusion line.

Our present results show clearly that this strategy is not acceptable for VPT incompatibility. Regardless of which line is brought closer to the patient (V or PT), the counts of particles ≥ 10 µm and ≥25 µm were significantly higher than with the standard set-up. For both set-ups, the particle peak 2 became higher and broader when this strategy was used; this would increase the risk of blocking the catheter or worsening the patient’s condition.

If the V infusion is close to the patient, the duration of particle peak 2 may be explained by the fact that even when the PT infusion is stopped, PT solution is still present in the tubing between the two infusions. 

Nevertheless, this strategy might be worth considering. Indeed, when the PT line was close to the patient, no visible particles were observed. Furthermore, peak 1 disappeared. Therefore, moving the two drugs further apart might reduce the extent of VPT incompatibility. 

Again, our results show that lowering the V concentration is better than using a multilumen infusion device.

Our study had a number of limitations. Firstly, the three main pharmacopeias (European, American, and Japanese) have not issued guidelines on the standardized dynamic counting of non-visible particles in parenterally administered formulations. The pharmacopeias have only issued requirements for the preparation of parenteral formulations and do not consider what is administered at the egress of the catheter. The pharmacopeias describe the presence of sub-visible particles in injectable medicinal products in general and parenteral formulations in particular. However, the pharmacopeias’ methods are only applicable to measurements under static conditions for unmixed injectable preparations and are, therefore, not suitable for preparations for continuous IV infusion. However, we selected the same sizes of sub-visible particles (>10 µm and >25 µm, which can possibly obstruct pulmonary and tissue capillaries) in our dynamic perfusion conditions. Static counts of particles in drug solutions had been described in the previous study. The results showed that the vancomycin solution was not compliant for particles > 10 µm [18]. Secondly, it is important to bear in mind that the results obtained were specific for VPT incompatibility at the chosen concentrations, without any other changes to the intravenous lines. It is highly possible that different results would be obtained with other drug concentrations and other clinical protocols involving a different number of drugs. However, our research methodology could be applied to other incompatibilities. Thirdly, we did not study the influence of the infusion set-up on the stability of the drug mass flow rates and the attainment of a steady state. These important variables are also conditioned by the total flow rate, which changes as a function of the infusion protocol. At last, we chose to perform our experiments at room temperature, even though the temperature is likely to influence the particle release. It would also have been interesting to study the relationship between the volume infused and the potentially infused particulate load.

## 4. Materials and Methods

### 4.1. Experiments, Devices, and Drugs

#### 4.1.1. Products and Medical Devices

The drugs, solvents, and medical infusion devices used are listed in Table 3 and Table S1. The two piperacillin/tazobactam generics (from Mylan and Panpharma) are identical (active pharmaceutical ingredients in sodium salt form) and have no excipients. The two Vancomycin generics (from Mylan and Sandoz) are in the form of vancomycin hydrochloride. Vancomycin Mylan has no excipients. Only Vancomycin Sandoz contains mannitol, sodium hydroxide, and hydrochloric acid as excipients. For both antibiotics, we do not know the active pharmaceutical ingredients supplier.

#### 4.1.2. Infusion Lines and Standard Operating Procedures

**Figure 6:**
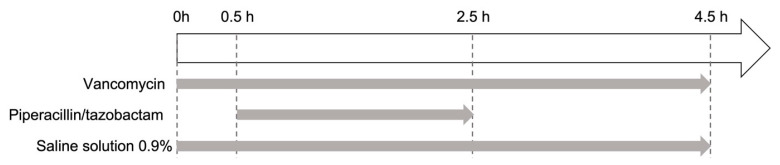
Timeline for the in vitro infusion of vancomycin and piperacillin/tazobactam.

In order to study the impact of the choice of infusion set-up on the risk of VPT incompatibility, eight infusion set-ups were studied at room temperature (n = 5). All the infusion durations in the study corresponded to the time from the start of V infusion to the end of V infusion. V and saline solution were infused continuously for 4.5 h. PT solution was infused for 2 h (from t = 0.5 h to t = 2.5 h) (Figure 6). For practical reasons, the duration of infusion was shortened from 9 h (the duration in the clinic) to 4.5 h in the laboratory. Nevertheless, the study focused on the critical period at risk of drug incompatibility during continuous infusions.

**Figure 7:**
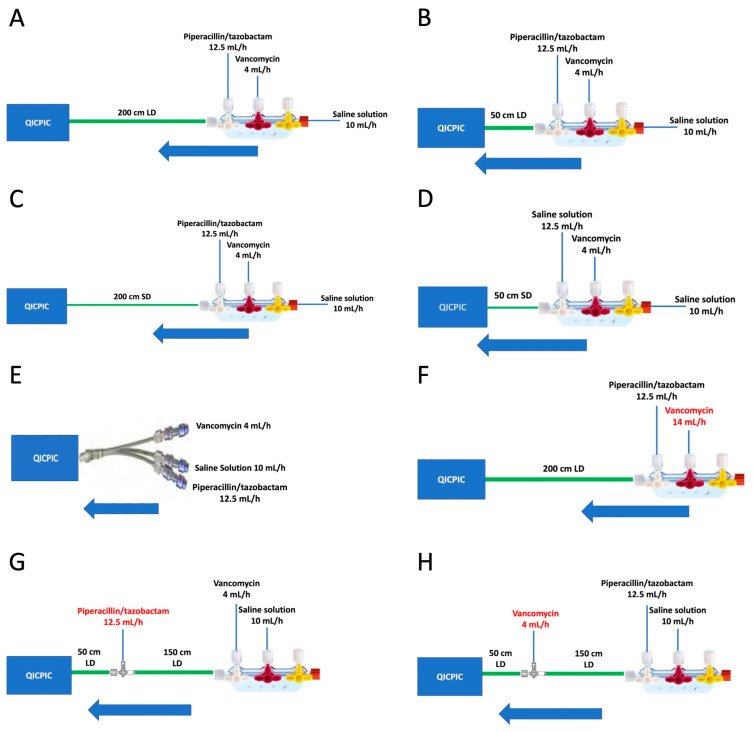
Representation of the standard manifold infusion set (200 cm tubing with 2.5 mm diameter) (**A**) and other infusion sets: 50 cm tubing with a 2.5 mm internal diameter (**B**), 200 cm tubing with a 1 mm internal diameter (**C**), 50 cm tubing with a 1 mm internal diameter (**D**), a multilumen device (**E**), the manifold infusion set with diluted V (**F**), the manifold infusion set with infusion of PT far from the manifold (**G**), and the manifold infusion set with infusion of V far from the manifold (**H**). The blue arrows represent the direction of infusion flow.

Seven set-ups (Figure 7B–H) were compared with a standard set-up designated as set-up A (Figure 7A). This standard set-up replicated the combined infusion of V and PT at standard doses, with a three-port manifold connected to a 200 cm extension set with a 2.5 mm internal diameter. A concomitant saline infusion was set up as volume support over a 4.5 h period (Table 4 and Figure 7). The flow rates, medical devices, V concentration (4 mL/h; 20.8 mg/mL), and PT concentration (12.5 mL/h; 80/10 mg/mL) corresponded to the usual concentrations used in intensive care units in France [18,28].

Seven additional infusion set-ups (B, C, D, E, F, G, and H) were evaluated. With the exception of set-up F, all the set-ups had the same drug flow rates and concentrations as set-up A. Set-ups B, C, and D were composed of a three-port manifold and tubing assemblies but differed from the standard set-up (A) with regard to the tubing’s length and/or internal diameter (Figure 7B–D). Two different lengths (200 cm or 50 cm) and two different internal diameters (2.5 mm (large Ø) and 1 mm (small Ø)) were tested. The common volume of each infusion set is described in Table 5. Set-up E included a multilumen device with a very low common volume. The common volume corresponds to the volume of the tubing within which the two drugs are in contact during the infusion. This common volume is represented by one or two green lines in the set-ups shown in Figure 7. For all infusion set-ups except set-up E, the common volume corresponded to the volume of the tubing or tubings at the manifold outlet. Additionally, set-up F had a lower V concentration (5.95 mg/mL) and a higher flow rate (14 mL/h), while the mass flow rate was the same as with the other set-ups without additional hydration (Table 4 and Figure 7F). The last two set-ups (G and H) had a longer distance between the two antibiotic infusions: either (i) the PT infusion was placed 150 cm downstream of the manifold (50 cm upstream of the infusion line’s outlet) (G) (Figure 7G) or (ii) the V infusion was placed 150 cm downstream of the manifold (50 cm upstream of the infusion line’s outlet) (H) (Figure 7H).

### 4.2. Instrument: Dynamic Particle Counts

A Qicpic dynamic image analysis device (Sympatec GmbH Inc., Clausthal-Zellerfeld, Germany) with a Lixell module (Sympatec GmbH Inc.) was used. A high-speed camera captured up to 500 images per second at a resolution of 1024 × 1024 pixels and was coupled to a frame rate of 10 Hz. Using Windox 5.0 software (Sympatec GmbH Inc.), the dynamic particle counter counted particles between 1 µm and 30 mm in size. The Lixell module was made up of end caps Luer locks. This enabled connection to the Qicpic apparatus. In the present study, the outlet tubing of the infusion set was directly connected to the Qicpic. Accurate measurements of the particle count were taken every five minutes throughout the infusion period. We recorded the total count of particles ≥ 10 µm and ≥25 µm throughout the 4.5 h infusion.

### 4.3. Statistical Analyses

The mean ± standard deviation total particle count (size: between 1 µm and 30 mm) during the 4.5 h infusion was represented graphically. The counts of particles ≥ 10 µm and ≥25 µm were analyzed as the median (range) in box-and-whisker plots and as the mean ± standard deviation in tables. All data were plotted and compared in two-tailed, non-parametric Mann–Whitney tests (GraphPad Prism software, version 6, LLC, San Diego, CA, USA). The threshold for statistical significance was set to *p* < 0.05.

## 5. Conclusions

Our results emphasized the complexity of drug incompatibility phenomena in medical infusion devices and showed that it is possible to reduce particulate contamination considerably under specific conditions. This work lays the foundations for the investigation of the occurrence of drug incompatibility as a function of the infusion device’s microfluidic characteristics.

## Figures and Tables

**Table 1:** Total particle count and the counts of particles ≥ 10 µm and particles ≥ 25 µm over the course of the infusion within peak 1, within peak 2, and within peaks 1 + 2 for set-ups A to H. The results are expressed as the mean ± SD (n = 5). The particle count in a given period is also expressed as percentage of the count over the total infusion period.

	Total Infusion Time			Peak 1			Peak 2			Peak 1 + 2		
Particles	Total	≥10 µm	≥25 µm	Percentage of Total Particles	Percentage of Particles ≥10 µm	Percentage of Particles ≥25 µm	Percentage of Total Particles	Percentage of Particles ≥10 µm	Percentage of Particles ≥25 µm	Percentage of Total Particles	Percentage of Particles ≥10 µm	Percentage of Particles ≥25 µm
Set-up A	1,679,849 ± 544,761	115,494 ± 21,315	20,469 ± 6023	84.2%	86.6%	94.0%	8.7%	8.7%	5.7%	92.9%	95.3%	99.8%
Set-up B	9,069,394 ± 4,145,341	671,673 ± 172,810	148,887 ± 74,396	12.0%	18.1%	22.7%	69.6%	85.1%	77.3%	97.9%	99.3%	99.9%
Set-up C	587,312 ± 205,362	30,830 ± 10,810	3738 ± 2405	66.5%	65.0%	70.8%	6.2%	12.5%	18.8%	72.7%	75.4%	89.6%
Set-up D	2,209,363 ± 842,171	169,728 ± 34,977	45,903 ± 9743	20.4%	18.3%	16.0%	73.4%	77.1%	83.6%	93.8%	95.4%	99.6%
Set-up E	3,703,771 ± 1,020,732	304,889 ± 81,586	71,841 ± 17,097	9.0%	8.9%	7.8%	82.3%	84.2%	87.1%	91.3%	93.1%	94.9%
Set-up F	64,300 ± 13,162	485 ± 127	5 ± 8	no peak			no peak			no peak		
Set-up G	4,405,799 ± 808,412	302,880 ± 109,452	34,592 ± 38,470	no peak			98.7%	98.2%	99.4%	98.7%	98.2%	99.4%
Set-up H	13,090,974 ± 4,070,835	1,306,725 ± 431,423	361,961 ± 154,142	13.5%	15.0%	14.9%	85.4%	84.7%	85%	98.9%	99.8%	99.9%

**Table 2:** Duration of peaks 1 and 2 in the various set-ups. The results are expressed as the mean ± SD, n = 5.

	Duration of the Peak (Minutes)	
Set-Up	Peak 1	Peak 2
A	7.0 ± 2.7	13.8 ± 7.5
B	9.2 ± 2.0	25.0 ± 4.5
C	5.0 ± 0.0	6.3 ± 2.5
D	5.0 ± 0.0	15.8 ± 2.0
E	6.0 ± 2.2	10.0 ± 0.0
G	-	36.3 ± 2.5
H	10.0 ± 0.0	500 ± 6.1

**Table 3:** The infused drugs and diluents used in the in vitro study.

Product	Pharmaceutical Company	Dose/Initial Concentration	Batch Number	Batch Expiry Date (Month/Year)
Vancomycin	Mylan (Morgantown, WV, USA)	1 g	B2407 B2422	06/2022 09/2022
	Sandoz (Vienna, Austria)	1 g	C0421 D0337	12/2023 07/2024
Piperacillin/tazobactam	Panpharma (Beignon, France)	4 g/500 mg	306725 306767	12/2023 02/2024
	Mylan	4 g/500 mg	18Y0758 18Y1943	10/2025 01/2026
Saline solution for injection	Baxter (Thetford, UK)	0.9%	21A23T4A 22F18T3B	12/2022 05/2025

**Table 4:** In vitro preparation of the drugs used in the eight infusion sets.

Drug or Injectable Product	Set-Up(s)	Reconstitution/Dilution Volume (0.9% Saline)	Container	Concentration (mg/mL)	Infusion Flow Rate (mL/h)
Vancomycin	A, B, C, D, E, G, H	48 mL q.s.	Syringe	20.8	4
	F	168 mL q.s.	Infusion bag	5.95	14
Piperacillin/tazobactam	A, B, C, D, E, F, G, H	50 mL q.s.	Syringe	80/10	12.5
0.9% saline solution	A, B, C, D, E, G, H	250 mL q.s.	Infusion bag	-	10
	F	-	-	-	-

**Table 5:** Length, internal diameter, and common volume of the tubing for each infusion set-up.

Set-Ups	Length of Tubing	Internal Diameter	Common Volume
A, F (200 large Ø)	200 cm	2.5 mm	9.82 mL
B (50 large Ø)	50 cm	2.5 mm	2.45 mL
C (200 small Ø)	50 cm	1 mm	1.66 mL
D (50 small Ø)	50 cm	1 mm	0.42 mL
E (multilumen)	-	-	0.06 mL
G, H	50 cm	2.5 mm	2.45 mL

## Data Availability

Data will be made available on request.

## Supplementary Materials

The following supporting information can be downloaded at: https://www.mdpi.com/article/10.3390/ph17091222/s1, Table S1: The medical devices used for preparation and infusion in the present in vitro study.
