# Supplementary material for: Evaluation of the Impact of Infusion Set Design on the Particulate Load Induced by Vancomycin–Piperacillin/Tazobactam Incompatibility
Source: Pharmaceuticals (Basel). 2024 Sep 17;17(9):1222. doi: 10.3390/ph17091222 (PMC11435023; doi:10.3390/ph17091222)
Supplement: Supplementary file 1 [file pharmaceuticals-17-01222-s001.zip › pharmaceuticals-3146556-supplementary.pdf]

Table S1: The medical devices used for preparation and infusion in the present *in vitro* study

Medical device	Producer	REF	Batch number	Batch expiry date (month/year)
**Two-port manifold + extension set 50 cm Ø 2.5 mm**	Cair LGL, Lissieu, France	RPB2320	16J10-T	09/2021
**Two-port manifold + extension set 200 cm Ø 2.5 mm**	Cair LGL, Lissieu, France	RPB2305	20K02-TQP1	10/2025
**Extension set 50 cm Ø 1 mm**	Vygon, Ecounen, France	1155.05	250419EK	04/2025
**Extension set 100 cm Ø 1 mm**	Vygon, Ecounen, France	1155.10	080720EK	07/2025
**Extension set 200 cm Ø 1 mm**	Vygon, Ecounen, France	1155.20	161018EK	10/2023
**Extension line for the infusion pump**	Fresenius Kabi, Bad Homburg, Germany	M46441000S	30043401	01/2026
**Multi-access infusion safety device EDELVAISS-CW3+**	Doran International, Toussieu, France	306.0923	30 22 07	07/2027
**50 mL Luer-Lok Syringe**	BD Plastipak, Madrid, Spain	300865	2011012	10/2025
**20 mL Luer-Lok Syringe**	BD Plastipak, Madrid, Spain	300629	2004266	03/2025
**Needles, 18G**	BD Microlance, Huesca, Spain	304622	210207	01/2026
